# Hemodynamic forces derived from cine cardiac magnetic resonance imaging: technical aspects, applications in various clinical scenarios, and in athlete's heart

**DOI:** 10.3389/fcvm.2026.1778061

**Published:** 2026-03-26

**Authors:** Dinara Jumadilova, Nurmakhan Zholshybek, Tairkhan Dautov, Gulmira Ayapergenova, Giovanni Tonti, Alessandro Salustri

**Affiliations:** 1 Department of Medicine, School of Medicine, Nazarbayev University, Astana, Kazakhstan; 2 Clinical and Academic Department of Radiology and Nuclear Medicine, Corporate Fund “University Medical Center”, Astana, Kazakhstan; 3 Department of Cardiovascular Disease, University G. D'Annunzio, Chieti-Pescara, Italy

**Keywords:** athlete's heart, cardiac magnetic resonance, global longitudinal strain, hemodynamic forces, left ventricular function

## Abstract

Recently, intraventricular pressure gradients, or hemodynamic forces (HDF), which are their global measure integrated over the left ventricular volume, have been proposed as a new concept capable of detecting subtle changes in left ventricular function. Thanks to a mathematical model, the analysis of routinely acquired cine cardiac magnetic resonance (CMR) images is now feasible without the need for contrast administration or 4D flow imaging, making it an attractive tool for the early detection and follow-up of left ventricular dysfunction. HDF derived from cine CMR images have been applied in normal subjects and in several pathological conditions, and the results of these studies confirm the feasibility and support the usefulness of this method in clinical practice. This review focuses on the technical aspects of cine CMR-derived HDF, emphasizing the need for precise image acquisition. Furthermore, we review the clinical relevance of HDF in various clinical conditions, illustrating their potential in the detection of cardiac diseases at an early stage, evaluation of medical/interventional treatment, and prediction of future cardiac events. Additionally, we report the results of HDF in athletes, where the HDF analysis is able to discriminate physiological adaptations from pathological cardiac remodeling and to document the effect of intense physical training. Future developments should include consistency of HDF parameters, and at this aim we have suggested a standardized approach for HDF analysis for clear interpretation and clinical use.

## 1 Introduction

Cardiac magnetic resonance (CMR) imaging is the reference method for assessing left ventricular (LV) morphology and function. Standard parameters, including LV volumes and ejection fraction, can be reliably derived from CMR images; however, these parameters have intrinsic pathophysiological limitations. To overcome these limitations, magnetic resonance feature-tracking based on post-processing methods has been proposed, which can resolve the analysis of myocardial deformation and derive strain parameters; however, the magnitude of myocardial strain is influenced by loading conditions (preload and afterload), heart rate, and chamber size and geometry (1).

Recently, intraventricular pressure gradients (IVPG) have been recognized as the determinants to propel the blood through the aorta and may offer insight into LV function. IVPG, generated by the combined effects of myocardial contraction and blood flow dynamics, pushes the incompressible blood that interacts with the LV walls and produces an equal and opposite force on the endocardium. These forces, which represent the global measure of the IVPG integrated over the LV volume, have been called hemodynamic forces (HDF). With this approach, the forces resulting from the adaptation of the left ventricle to different pressure/volume overload can be displayed, and thus LV dysfunction is detected at an early stage. Eventually, the highly sensitive nature of HDF as a marker of myocardial function makes it ideal for monitoring the progression of heart disease, evaluating the impact of treatment, and prognosticating future cardiovascular events.

While other reviews on this field have been recently published (2–4), in this article we will focus on HDF derived from cine CMR. In particular, we aim to describe the technical aspects of cine CMR image acquisition, required for achieving reliable results. In addition, the results of this method applied in several clinical conditions are reported, with a special emphasis on the information provided in athletes.

## 2 Cine CMR acquisition methods

Various acquisition strategies can be applied to cine CMR. The standard method is a segmented breath-held retrospectively ECG-gated acquisition (full sampling method) that typically utilizes balanced steady-state free procession sequences. The high signal-to-noise ratio (SNR) and the excellent blood-myocardium contrast result in high quality images, however the method is time-consuming (5).

Reduction of acquisition time can be achieved with parallel imaging, which allows for acceleration at factors of 2–4. This needs prior information for reconstruction—either coil sensitivity maps in the SENSE (Sensitivity Encoding) method, or kernel weights estimated from auto-calibration signals in the GRAPPA (Generalized Autocalibrating Partially Parallel Acquisition) method. Both methods of parallel imaging suffer from lower SNR (about one third) compared to standard sampling (6–8).

Another approach to shorten the acquisition time is the real-time (RT) approach, in which individual sequences are collected rapidly in one heartbeat to eliminate the need for breathholding and ECG-gating and potentially to avoid motion. This means patients with arrhythmias can be examined without artifacts. SNR in RT images is significantly lower than in standard acquisition; therefore, RT imaging is combined with parallel imaging, though the achieved SNR is still inferior to non-accelerated imaging (9). In our center, on Siemens 1.5 T Magnetom Avanto, PI is incorporated into scanning sequences, and real-time imaging is used for pediatric patients who are unable to cooperate with breathholding.

A more advanced method is compressed sensing (CS) which exploits the potential of CMR imaging to represent data based on fewer points in certain domains within a single breath-hold while maintaining information without significant loss due to iterative, non-linear reconstruction. This allows reduction in scan time making it advantageous for imaging of patients who are unable to tolerate multiple breath-hold instances during standard cine acquisition. The quality of CS images is comparable to segmented acquisition, making it convenient in patients with arrhythmias. However, due to undersampling of k-space, CS is prone to noise and aliasing artifacts. The acceleration factor selected is a trade-off point between time reduction and image fidelity, with a recommended acceleration factor at less than 8. Thus, CS has high SNR with short acquisition time among all methods (10–12).

## 3 Technical aspects of cine CMR image acquisition

According to the international recommendations, the standard CMR acquisition protocol includes several sequences (5, 13). In our Center, cine images are sliced at 8 mm thickness with a 2 mm interslice gap, yet variations may exist among centers, such as 6–7 mm thickness with a 3–4 mm gap. The flip angle in our examinations is 55 degrees, the repetition time 34.68 ms, and the echo time 1.22 ms.

**Figure 1:**
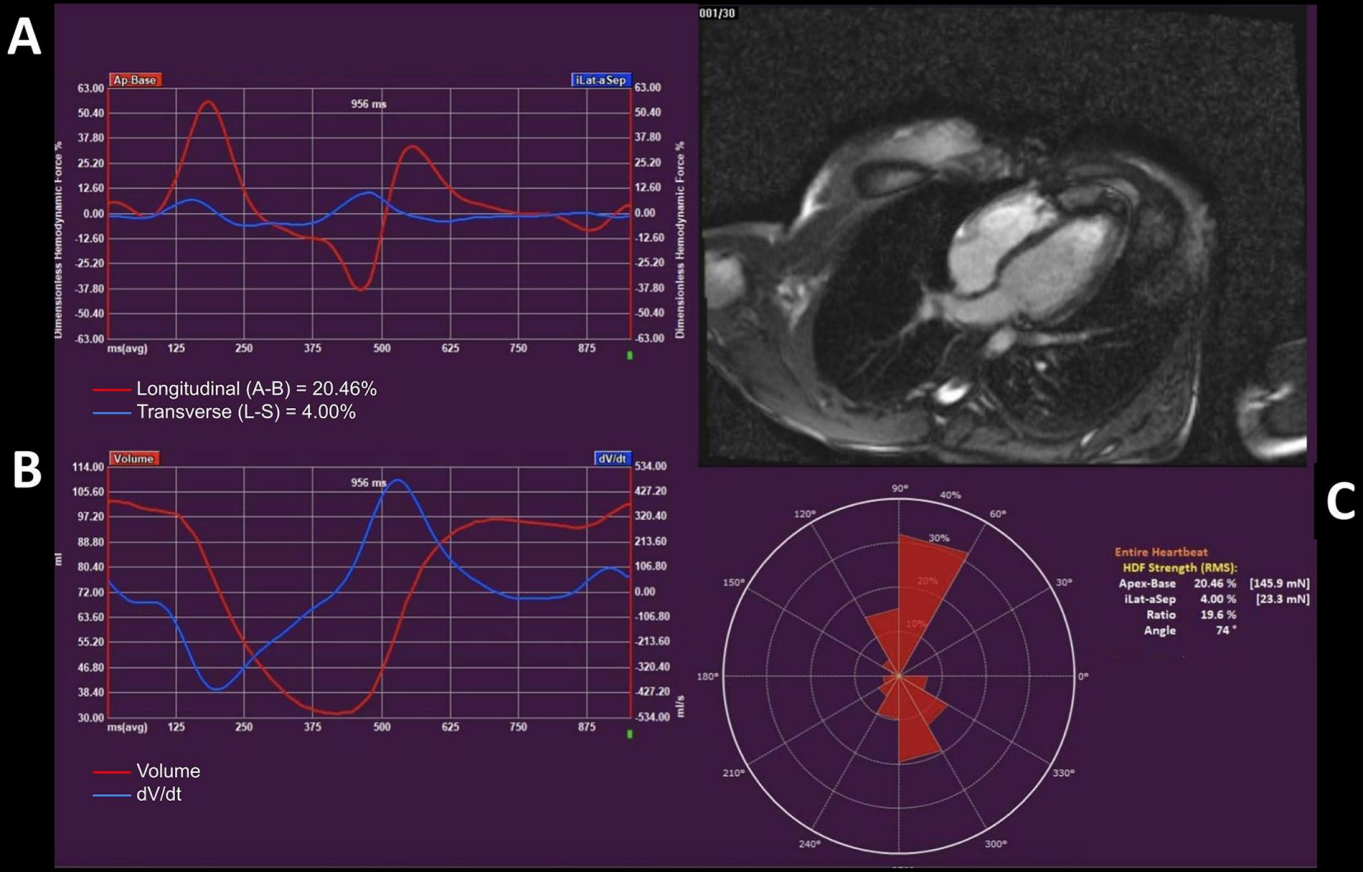
Panel **(A)** graph: hemodynamic forces (HDF) in a healthy subject. The red line represents the longitudinal HDF that are the most consistently reproducible and detectable ones. Positive deflections indicate an apex-to-base direction, while negative deflections indicate a base-to-apex direction of the intraventricular pressure gradients. The blue line represents the transversal HDF that in normal subjects have a small amplitude. Significant transversal forces may indicate underlying cardiac disorders and should be properly investigated. Panel **(B)** graph: The two curves represent the changes of the LV volume over time (in red) and the rate at which the LV volume changes over time (dV/dt) (in blue). Panel **(C)**: Diagrammatic representation of the average angle of the forces (in degrees) and numerical values of HDF throughout the entire heartbeat.

The assessment of HDF requires proper acquisition of cardiac images in three long-axis (LAx) cine views. While LAx views are appropriate for global longitudinal strain, short-axis views are utilized in case of evaluation of global circumferential and radial strains. With advanced software post-processing of LAx images, feature-tracking of cardiac contours is feasible, and parameters of myocardial deformation are derived. Then, from strain assessment, HDF can be calculated through measurement of the mitral and aortic valve areas. Once measured, the software immediately displays the pattern of HDF within the left ventricle throughout the cardiac cycle (Figure 1).

**Figure 2:**
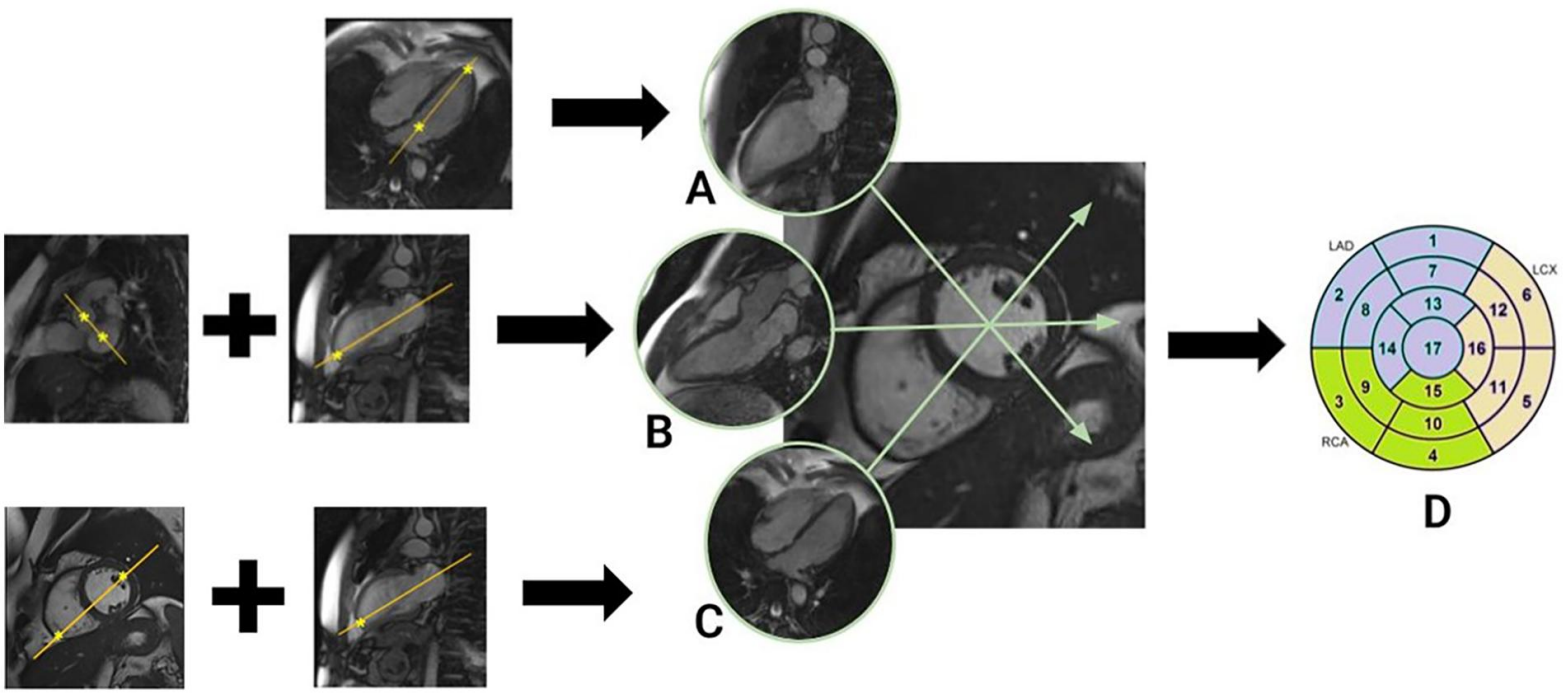
The spatial relationship of the cardiac long axes with its short axis. Three views are acquired: **(A)** Two-chamber: from the pseudo-four-chamber view (right), the operator identifies the midpoint of the mitral valve and the cardiac apex (asterisks). A cut plane crossing these points will provide the two-chamber LAx view; **(B)** Three-chamber: from the basal pseudo-short-axis view, the operator selects the mid-points of the mitral and aortic valve (mid, asterisks), while in the two-chamber LAx view, the cardiac apex is detected (right, asterisk). From these three points, the plane of the three-chamber LAx view is obtained; **(C)** Four-chamber: from the midpoint of the mitral valve to the apex on pseudo-two-chamber view; and, from the free right ventricular angle to the antero-lateral papillary muscle of the left ventricle on pseudo-short-axis view. With this approach, the left ventricle can be divided into 17 segments that can be represented in a bull's eye format **(D).**

The feasibility and robustness of HDF are highly dependent on the quality of image acquisition, i.e., precise planimetry. With LAx views, the inclusion of the apex into the slice to be analyzed is crucial to obtain the full longitudinal axis of the left ventricle. To acquire a two-chamber view, the operator has to place the plane axis on an axial scout (pseudo-four-chamber) image through the midpoint of the mitral valve and the apex (Figure 2, panel **A**). For a three-chamber view, slicing should be parallel to the plane of three points, namely the midpoint of the mitral and aortic valve (the pear-shaped view in the basal short-axis slice), and the cardiac apex (in the two-chamber LAX view) (Figure 2, panel **B**). The four-chamber view is planned on pseudo-two-chamber by placing the points at the midpoint of the mitral valve and the apex, at the same time on pseudo-short-axis view—connecting the free right ventricular angle with the anterolateral papillary muscle of the left ventricle (Figure 2, panel **C**). From the three LAx views, the LV myocardial walls can be visualized 360^°^ (“bull's eye”), which provides a comprehensive assessment of all the LV wall segments (Figure 2, panel **D**).

**Figure 3:**
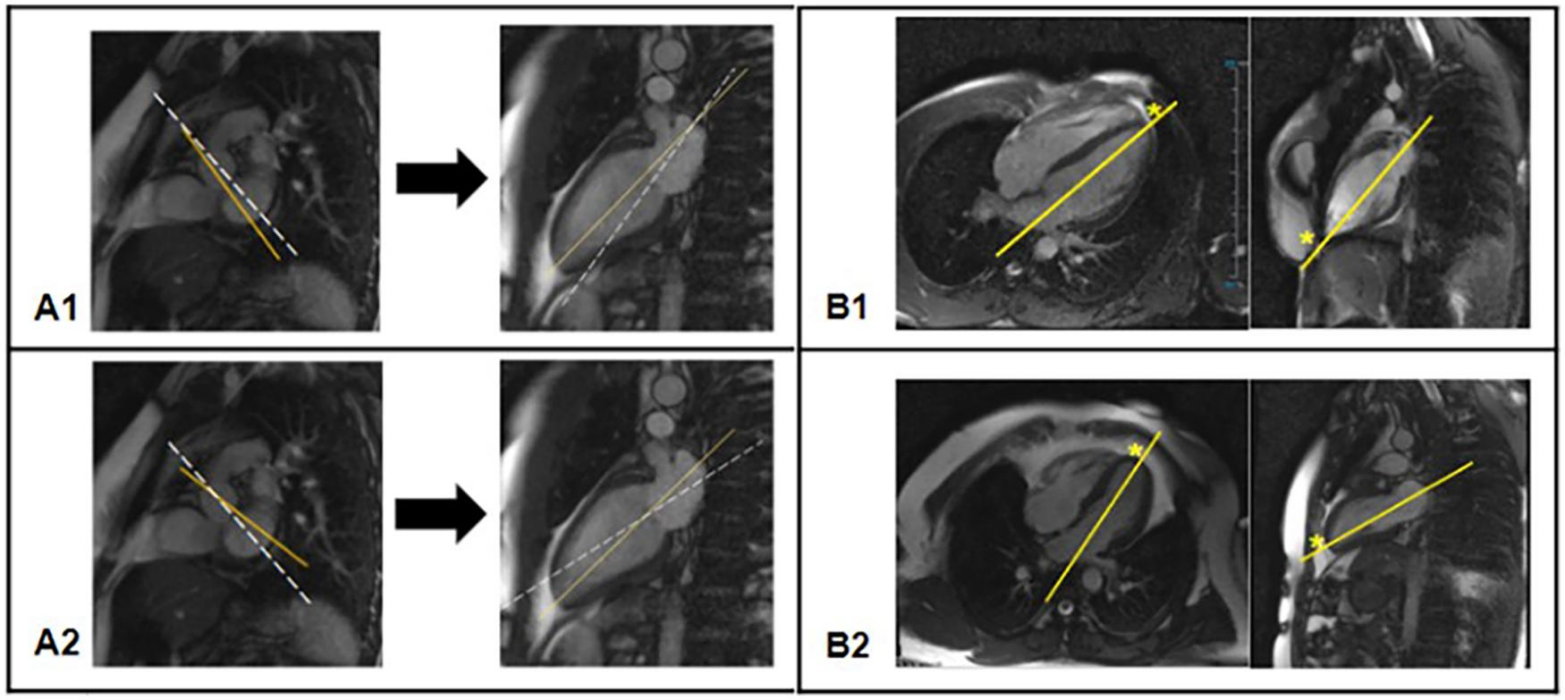
Panels **(A1,A2)**: slightly skewed long axis lines on basal short axis views. Dashed lines represent the intended plane of scanning on short-axis views, and the resulting plane on the two-chamber views, which lies far from the apex. Yellow lines demonstrate the actual planes of scanning to include the apex. Panel **(B)**: Four- and two-chamber long axis views of a heart with acute **(B1)** and rounded **(B2)** apex.

Technical challenges may occur during image planning. Reaching the exact midlines on both short-axis and two-chamber views when placing the plane axis for the acquisition of the three-chamber view can be challenging. In such a case, a compromise should be sought between both views to maximize the accuracy, and usually the line at the basal short axis level can be slightly skewed to include the apex on the two-chamber view. This does not have a significant impact on the quality of acquired three-chamber views for the evaluation of myocardial strain and HDF (Figure 3, panels **A1** and **A2**).

Although the planimetry for the two- and three-chamber LAx views appears quite simple (13), the cardiac anatomy among individuals varies greatly in terms of apex position. By definition, the cardiac apex “is the apex of the conical left ventricle” (14); however, the shape of the left ventricle may not appear as a sharp cone. Moreover, in some cardiac diseases (such as LV hypertrophy), the cardiac apex becomes rounded, and even in normal subjects the heart may have an anatomically rounded shape. Therefore, finding an acceptable definition for the radiological apex of the heart can be challenging. In practice, when the apex is acute, the plane of scanning is defined as shown in Figure 3, panel **B1**. With a rounded (blunted) shape of the cardiac apex, the operator should place the target point at the midpoint of the apical segment of the left ventricle to avoid foreshortening in estimation of the blood flow forces within the LV chamber (5, 15) (Figure 3, panel **B2**).

Another challenge may be faced in the two-chamber view in case of hearts with a concave inferior wall, where the mitral valve midpoint and the apex are connected in such a way that the plane of the four-chamber crosses the myocardium and not the LV cavity. Therefore, for the analysis of LV HDF, positioning the plane slightly higher in relation to the apex is practical to avoid crossing the LV inferior wall.

Similar to echocardiography, when the cine CMR image quality is suboptimal and the tracing cannot be precisely done on more than two myocardial segments the calculation of global longitudinal strain is hampered. Moreover, motion, susceptibility, and other artifacts may interfere with the field of scanning and thus prevent adequate acquisition. In such a case, the presence of the views from other planes allows to fill in the segmental data gap and perform the analysis.

## 4 HDF derived from CMR imaging

Historically, HDF analysis has been performed using 3D time-resolved CMR imaging (4D flow), which is considered the reference method (4, 16). Benefits of the 4D flow include a small sensitivity to errors in ventricular contouring, accuracy and precision for HDF, and the capacity to adequately depict the flow field even in distorted left ventricles. However, several technical factors have limited the clinical application of this technique (17–19). Recently, thanks to a mathematical model based on the first principle of fluid dynamics, endocardial dynamics are transformed into flow forces, which allows analysis of routinely acquired images irrespective of the availability of 4D flow (20). This method relies exclusively on the position and velocity of the endocardium and the valvular planes; thus, routine cine CMR sequences can be used to calculate HDF, which makes the analysis of HDF an attractive tool in clinical practice. The relative characteristics, advantages, and disadvantages of 4-D flow and cine-CMR techniques are summarized in Table 1.

**Table 1:** Main differences between 4D flow and сine CMR.

Features	4D flow CMR	Cine CMR
Initial purpose	Visualization and quantification of multidirectional blood flow	Visualization of cardiac anatomy and wall motion
Dimensions	3D + time (4D) = anatomy and velocity	2D or 3D + time (cine loops)
Image acquisition	Phase-contrast MRI with velocity encoding in 3D across the cardiac cycle Single, long acquisition (5–25 min) ECG/respiratory gated	Multiple short breath-holds heart heartbeats assembled into a loop
Measurements	Advanced	Standard
Flow information	Direct, multidirectional	Indirect, limited
Advanced hemodynamics	Complex hemodynamic forces	Not possible
Applications	Complex flow visualization	Routine cardiac function
Post-processing	Complex and time-intensive Requires dedicated software and expertise	Contour definition
Key advantages	Comprehensive hemodynamic quantification Retrospective flow analysis Multidirectional coverage	Fast and robust Widely available High temporal resolution
Limitations	Long acquisition time Lower spatial and temporal resolution One velocity encoding value • Aliasing • Low velocity-to-noise ratio Motion/turbulence artifacts Time-consuming post-processing Expensive Limited availability	Limited to motion/morphology Patient's cooperation (repetitive breath-holding) Sensitive to arrhythmias and motion artifacts Lower spatial resolution

There is scarce evidence in the literature in regard to comparison of HDF computation between cine CMR and 4D flow. In his fundamental article on the method, Pedrizzetti et al. validated the mathematical model based on endocardial surface reconstructed from the three standard long-axis projections against 4D flow MRI in 15 subjects (21). The results showed a good correlation (r = 0.88) and non-bias (best fit: *y* = 0.98 *x*) estimation of the longitudinal HDF. The correlation was good for the transversal force as well (*r* = 0.84), although the model gives a small underestimation of the RMS value (best fit: *y* = 0.87 *x*).

## 5 From cine CMR to HDF time curve

After the acquisition of a four, two, and three-chamber view at cine CMR, HDF can be computed. The calculation requires the velocity over the endocardial boundary and the blood velocity across the valves. The former is derived from the same feature tracking used for strain analysis. The latter is calculated from the volumetric changes of the left ventricle and the mitral and aortic valve area, using the conservation of mass principle. The HDF curve obtained represents the integral over the LV blood volume of the force per unit volume (pressure gradient), and the result is a force measurable in Newton (N), calculated thanks to the equation:

F(t)=ρ∫S(t)[x(∂v∂t⋅n)+v(v⋅n)]dS,

where **v**(x, t) is the fluid velocity vector field measured at fixed points **x** at time **t, S(t)** is the closed surface bounding the volume, and **n** is the outward unit normal vector. In this way, the force associated with a fluid volume can be evaluated from measurements carried out at the boundaries of the volume regardless of flow phenomena developing inside. Finally, the three-dimensional surface is reconstructed by combining the three long-axis views, and parameters similar to 4D flow are obtained.

For easy comparison between subjects with different LV sizes, values are normalized by the LV volume, which results in an acceleration (cm/sec^2^) and is finally expressed as a percentage of gravity acceleration (dividing by 981 cm/s^2^). Thus, units are dimensionless (%). We could argue that a dilated left ventricle may result in a reduction of HDF just because of the high value (volume) in the denominator of the formula. However, this is unlikely since volume is present in the nominator too (in fact, the force is an integral over the volume). Therefore, the impact of LV normalization on the HDF value is not relevant. More details on the mathematical model applied for HDF assessment can be found in Pedrizzetti (20).

**Figure 4:**
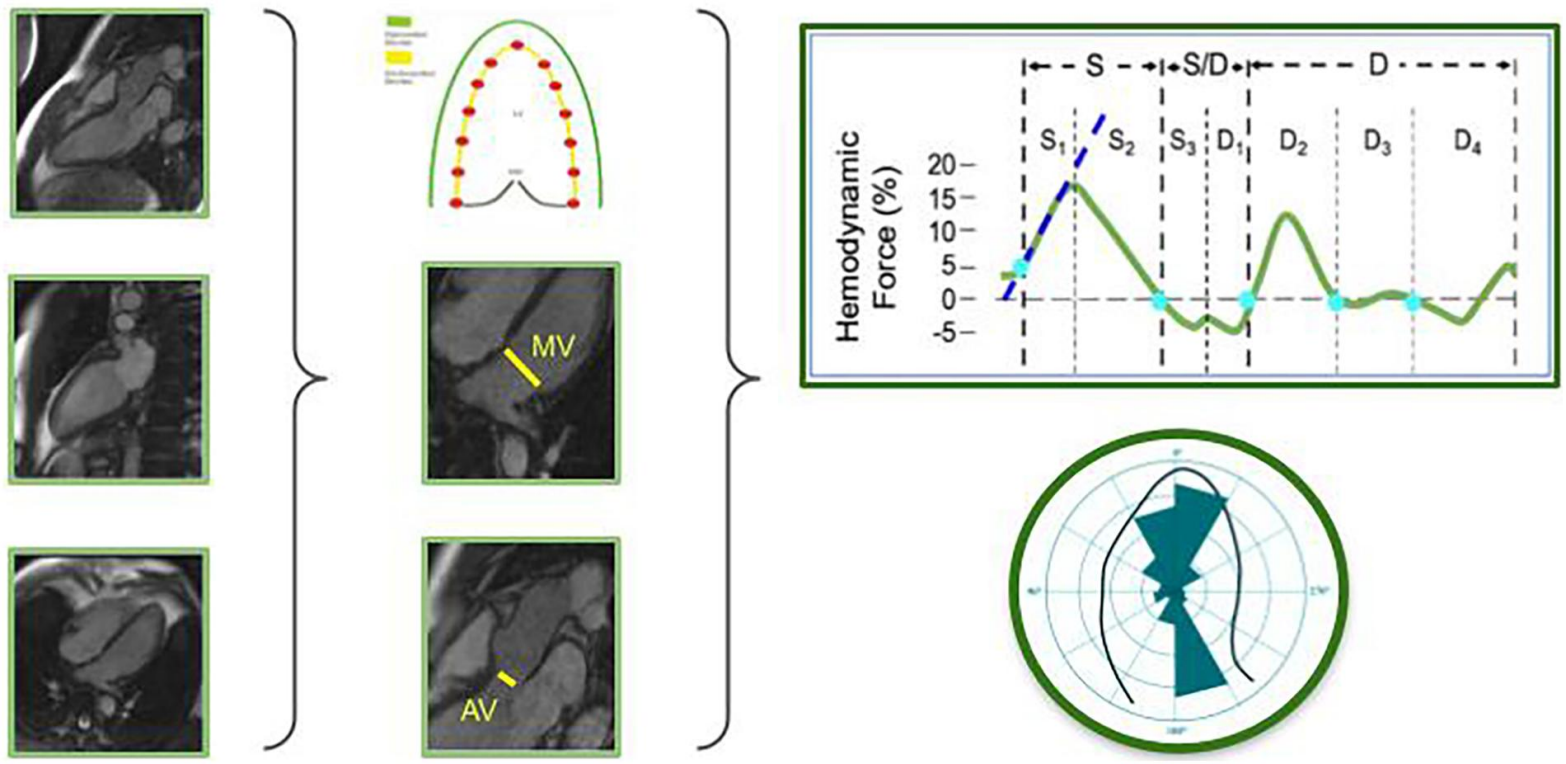
Flow chart of the steps required for HDF analysis. Images are acquired using non-contrast CMR. Three apical views are selected and the endocardial border is detected. Tracking of the endocardial motion provides strain assessment. Aortic and mitral valve areas are calculated from the relative valve diameters. Finally, a mathematical model based on the first principle of fluid dynamics and mass conservation principles allows for HDF curve assessment and display. AV, aortic valve; CMR, cardiac magnetic resonance; HDF, hemodynamic forces; MV, mitral valve. Adapted with permission from the Figure previously published by the author of this article (22), © 2024 the authors (CC BY NCND).

Normal HDF within the left ventricle are mainly distributed along three directions: (1) apex-to-base (longitudinal HDF); (2) septal-to-lateral (transverse HDF); (3) inferior-to-anterior. Usually, only the longitudinal and transverse HDF are displayed, with the former being the most relevant in normal subjects. In fact, vectors of blood-driven pressure within the left ventricle are typically oriented along the apex-to-base axis and ideally the ratio of longitudinal force to total force should be 100%, whereas an increase in the transverse force represents cardiac dyssynchrony and dysfunction. Values above the zero line indicate intraventricular pressure gradients with an apex-to-base direction, while values below the zero line represent gradients with an opposite (base-to-apex) direction. The HDF curve can be analyzed, and the value corresponds to the mean amplitude of the forces throughout the cardiac cycle, which is expressed as root mean square (RMS). Moreover, the curve can be divided into several phases of the cardiac cycle, including systolic ejection, systo-diastolic transition, and early and late diastolic filling. Peak value, slope, duration, and area under the curve (AUC) can be calculated for each cardiac phase (22). Figure 4 represents the steps from CMR acquisition to HDF analysis.

We have assessed the inter- and intra-observer variability of HDF parameters derived from cine CMR (15). Our results showed a high inter- and intra-observer reliability, both for the longitudinal and the transversal HDF. In our center, HDF are analysed by physicians with 7–10 years of experience in cardiac imaging, expertise with the imaging software, and regular joint reading sessions. Our results are in agreement with those published by Lange et al. (23) which shows excellent inter- and intra-observer reproducibility for the HDF parameters throughout the entire cardiac cycle, and lower variation for the systolic compared to the diastolic parameters.

## 6 Clinical applications of HDF

Clinical studies on HDF derived from cine CMR are presented in Table 2. In patients with heart failure, HDF was significantly lower when LV ejection fraction was preserved compared to a control (healthy) group and stepwise lower in cases of heart failure with preserved, mid-range, and reduced LV ejection fraction (24). Similar results were found in patients with heart failure with preserved ejection fraction, where longitudinal HDF was impaired compared to non-cardiac dyspnoea. In this group of patients, impaired peak systolic HDF was associated with cardiovascular hospitalization (HR 0.95, *p* = 0.016) (25).

**Table 2:** Studies on HDF derived from cine CMR imaging.

Author (Ref)	Study population	Parameters	Findings (as reported in the abstract of the article)
Lapinskas et al. (24)	Heart failure	Average amplitude Peak values	LV HDF may significantly improve the detection of early myocardial systolic dysfunction when volumetric and deformation cardiac measures are still intact
Backhaus et al. (25)	HFpEF	Average amplitude Systole Systolic-diastolic transition	Assessment of HDF indicates impairment of LV systolic ejection force in HFpEF, which is associated with cardiovascular events
Vos et al. (26)	Pre-capillary pulmonary hypertension	Systolic ejection Systo-diastolic transition E-wave deceleration A-wave acceleration	Pre-capillary pulmonary hypertension impacts LV function by altering diastolic function, demonstrated by an impairment of left atrium phasic function and LV IVPG analysis
Loke et al. (27)	Repaired tetralogy of Fallot (RTOF)	Amplitude Impulse Angles	RTOF patients have abnormal diastolic HDF that is correlated to pulmonary regurgitation, right ventricular function, exercise capacity, and vorticity
Shao et al. (28)	T2 DM	Entire cardiac cycle Longitudinal Transversal	HDF analysis may be a potential early marker of subclinical myocardial dysfunction
Filomena et al. (30)	Post-MI	Average amplitude	Misalignment of diastolic HDF after STEMI is associated with adverse LV remodeling after 4 months
Konijnenberg et al. (31)	Post-MI	Entire heart beat (longitudinal, lateral, ratio Systolic ejection Peak systolic force Systolic–diastolic transition Systolic slowdown Diastolic suction E-wave deceleration A-wave acceleration	CMR-derived LV IVPG are univariably associated with MACE However, LV IVPGs do not add prognostic value to LV ejection fraction and LV GLS
Vos et al. (32)	Dilated cardiomyopathy	IVPG	In the absence of pressure reversal, lower systolic ejection force, E-wave decelerative force, and overall LV IVPG are powerful predictors of outcome, independent of clinical and imaging parameters
Lapinskas et al. (33)	Chemotherapy	Entire cardiac cycle (longitudinal) Systole (longitudinal) Diastole (suction)	Despite no further significant decline in LVEF and GLS after 3 months of anthracycline therapy, HDF continue to decrease, reflecting subtle changes in LV preceding overt functional impairment Higher sensitivity of HDFs as biomarkers of cardiotoxicity of cancer-related therapy
Monosilio et al. (34)	Athletes	Entire cardiac cyle Systole Diastole Longitudinal Transversal Ratio	No significant differences among athletes of various disciplines Female athletes had higher EF and GLS than male ones, while male athletes showed slightly higher systolic component of longitudinal strength
Jumadilova et al. (35)	Athletes, hypertensive patients	Entire cardiac cycle (longitudinal, transverse, ratio) Slope of systolic ejection Systolic phase Suction Early filling Atrial thrust	HDF allows distinction between the hemodynamic patterns of athletes and patients with hypertension
Yang et al. (39)	Healthy volunteers	Entire heartbeat Systole Diastole Systolic/diastolic transition Diastolic deceleration Atrial thrust	The study provided comprehensive normal values of HDF assessments with specific age and sex stratification
Airale et al. (46)	Non-ischemic left ventricular cardiomyopathy	Clustering of longitudinal and transversal HDF	Analyzing both longitudinal and transversal HDF throughout the cardiac cycle enables the identification of distinct phenotypes with prognostic value beyond EF and LGE in non-ischemic LV cardiomyopathy

AUC, area under the curve; CMR, cardiac magnetic resonance; GLS, global longitudinal strain; HDF, hemodynamic forces; IVPG, intraventricular pressure gradients; LGE, low gadolinium enhancement; LV, left ventricular; MACE, major adverse cardiac events; RMS, root mean square.

In patients with precapillary pulmonary hypertension, Vos et al. (26) found that, compared to healthy volunteers, diastolic function was impaired before the LV ejection fraction starts to lower, as suggested by impaired diastolic suction and E-wave decelerative HDF.

Diastolic HDF amplitude was significantly increased in patients with repaired tetralogy of Fallot compared to controls as a consequence of chronic pulmonary regurgitation (27). HDF amplitude and impulse in the diaphragm-right ventricular outflow tract direction correlated well with percent of pulmonary regurgitation. In addition, VO_2_ max correlated with lateral-to-septal HDF impulse. Of note, when compared, cine-derived HDF and 4D flow-derived HDF in this category of patients had no significant measurement bias by Bland-Altman analysis.

In patients with type 2 diabetes mellitus, no differences in LV function and myocardial deformation were detected (28). In contrast, the transversal HDF was significantly higher compared to healthy individuals and tended to increase throughout the course of the disease as a sign of further abnormality.

Left ventricular remodeling after aortic valve replacement was studied by Rank et al. (29) during 10 years of follow-up. The results indicate that patients with aortic regurgitation had higher HDF compared to patients with aortic stenosis before valve replacement. In patients with aortic stenosis HDF remained stable over time, while patients with aortic regurgitation exhibited a decreased HDF value after 1 year and remained stable afterwards, which might explain the progressive deterioration of functional parameters and clinical status after valve replacement in patients with aortic regurgitation. Left ventricular remodeling was also evaluated in 49 patients with ST-elevated myocardial infarction (STEMI) (30). Those with an adverse LV remodeling at 4-month follow-up had lower systolic transversal HDF and higher diastolic transversal-to-longitudinal HDF ratio. In the multivariable logistic regression analysis, the diastolic transversal-to-longitudinal HDF ratio remained the only independent predictor of adverse LV remodeling [OR: 1.1 (1.01–1.2), *p* = 0.04].

As part of a large multi-centre registry, Konijnenberg et al. (31) analysed cine CMR performed in 307 patients within 14 days after STEMI. Their results suggest that CMR-derived HDF are invariably associated with major adverse cardiovascular events at a median follow-up of 9.7 years, and this association remains significant after adjustment for common clinical risk factors and measures of infarct severity. The prognostic value of HDF was also assessed in patients with dilated cardiomyopathy (32). A temporary LV IVPG reversal during systo-diastolic transition was observed in one third of the patients, and in 14% this led to a reversal of blood flow, which predicted outcome corrected for univariable predictors [HR = 2.57 (1.01–6.51), *p* = 0.047]. In the absence of pressure reversal, lower systolic ejection force, E-wave decelerative force, and overall LV IVPG were powerful predictors of outcome, independent of clinical and imaging parameters.

Patients with breast cancer are at risk of chemotherapy-induced cardiotoxicity. In patients receiving doxorubicin-based chemotherapy, cine CMR at baseline, 3-month, and 6-month follow-up revealed a progressive deterioration over time of the longitudinal HDF, the systolic HDF, and the diastolic suction (33). In contrast, both LV ejection fraction and GLS were significantly lower at 3-month follow-up, but remained within the normal range, and no significant changes were observed between the 3-month and 6-month follow-up.

In summary, the results of these studies suggest that HDF analysis is a potential early marker of subclinical myocardial dysfunction and may provide additional prognostic information.

## 7 HDF in athletes

HDF could provide new insights into the complex cardiac mechanics in athletes. At this aim, HDF were analysed in 320 Olympic athletes (skill, power, endurance, mixed) (34). As expected, endurance athletes showed the highest level of cardiac remodelling in terms of LV end-diastolic and end-systolic indexed volumes. No differences were found between athletes of different sports categories in terms of HDF measured throughout the entire cardiac cycle, in systole, and in diastole. Moreover, there were no differences in HDF longitudinal components between male and female athletes. These findings suggest that sports-related remodelling does not negatively affect cardiac function.

HDF can be clinically relevant for differential diagnosis between extreme remodelling due to exercise training and the initial stages of cardiomyopathies. At this aim, HDF were analysed in a group of athletes and compared to patients with hypertension (35). The results showed higher HDF in the first part of systole among athletes, represented by a steeper and higher slope of ejection. Athletic hearts also had shorter and less negative AUC of elastic rebound, which implied less time and energy expenditure to recover from preceding systolic deformation compared to stiffer hypertensive myocardium. Unlike in athletes, hypertensive hearts demonstrated abnormal suction, indicating inadequate untwisting in early diastole, while their phase of atrial thrust was significantly higher than in athletes, highlighting the importance of active LV filling. These findings translate into a better efficiency in myocardial work of athletic hearts in comparison with hypertensive patients. In the same study (35), HDF in endurance vs. strength athletes were compared. No significant difference in HDF along the entire heart beat were found, however analysis of single phases of the cardiac cycle showed that strength athletes have a shorter duration of the first phase of the systole and of the systolic impulse. These findings can be explained by the different modalities of generation of IVPG in the two types of exercise, and suggest that strength athletes need less time to reach the systolic peak and generate the same level of hemodynamic work. Additionally, the early filling of the left ventricle was lower in endurance athletes compared to strength athletes, which can be explained by the higher LVMi and LVEDVi in endurance athletes, which may affect the LV untwisting and filling.

Finally, the effect of intense physical training on HDF parameters in a group of endurance athletes was assessed by cine CMR performed off-season and at peak training (36). Interestingly, we found that intense physical training in endurance athletes is associated with changes in the HDF of the first part of the systole and of the elastic rebound, independent of changes in strain or LV structure. This approach might be useful to evaluate the effects of intense physical training and possibly to individualize the work training schedule for individual athletes.

Energetic parameters have also been studied in athletes by 4D flow cardiac MRI. Significant differences in kinetic energy fluctuation and vorticity fluctuation measured from the HyperDoppler analysis were observed in triathlon athletes compared to healthy subjects and sedentary groups. A distinctive trend was identified for the Energy Dissipation parameter in triathletes in the absence of geometrical modifications of the flow pattern. This aspect can suggest an independent marker of an athlete's heart (37). Moreover, compared to normal subjects and patients with arrhythmogenic cardiomyopathy, lower kinetic energy and shear stress have been documented in the right ventricular outflow tract of athletes, which might be explained by a higher diastolic compliance of the right ventricle (38). These emerging indicators may serve as sensitive markers of subtle changes within the cardiac chambers, however sophisticated elaboration in the postprocessing analysis represent the main disadvantage for an imminent significant diffusion.

## 8 Current gaps in research

Reference values for HDF are still scanty, however, like strain, normal values are age- and gender related (39). Ethnicity might add another potential layer of complexity, as reported in a large study on HDF in normal subjects using transthoracic echocardiography as an imaging modality (40). Finally, we have recently demonstrated that HDF values are significantly different according to the imaging modality applied (CMR vs. echocardiography) (41). Therefore, using the same imaging modality is mandatory for serial assessment of HDF.

Currently, there are gaps in the field related to HDF analysis. Despite the fact that the method has been used in various clinical scenarios, the parameters are not standardized yet. HDF values can be measured throughout the cardiac cycle, which provide insight into overall myocardial function, and during selected phases of the cardiac cycle (22). Some parameters describe the amplitude of the forces (peak values, RMS, impulse), while others describe their orientation (ratio, angle). Clearly, there is a lack of consistency regarding the definition of the cardiac phases and the appropriate time intervals for HDF measurements. Based on Pedrizzetti et al. (40), we recommend a systematic analysis of the whole heartbeat, and of systolic and diastolic phase alone. Table 3 represents the most relevant parameters that should be reported for each cardiac phase. This approach would help in standardization of the method and give the opportunity to prioritize analysis of a specific cardiac phase according to the clinical questions.

**Table 3:** Hemodynamic force parameters (modified from Pedrizzetti et al., EHJ-CVI 2025).

Phase	#	Name	Brief description	Formula
Whole heartbeat	1	Amplitude [%]	Root Mean Square (RMS) of the longitudinal force during the entire heartbeat. Overall strength of the force exchanged between blood and tissue.	Summation over the entire heartbeat
	2	Alignment [%]	Mean Alignment of the force vector. Ratio of longitudinal force to total force (100% is ideal)	Summation over the entire heartbeat
Systole	3	Sys-Amplitude [%]	RMS of longitudinal force in systole	Like (1), summation limited to systole
	4	Sys-Alignment [%]	Alignment of force in systole	Like (2), summations limited to systole
	5	Sys-Peak [%]	Maximum value of the systolic force	
	6	Sys-Impulse [%]	Time integral (Area under the curve, AUC) of the positive force in systole (with time normalized to heartbeat to maintain the same unit)	summation limited to that phase of systole
Diastole	7	Dia-Amplitude [%]	RMS of longitudinal force in diastole	Like (1), summation limited to diastole
	8	Dia-Alignment [%]	Alignment of force in diastole	Like (2), summations limited to diastole
	9	Dia-Suction [%]	AUC of negative force from late-systole through early-diastole	Like (6), summation limited to that phase of diastole
	10	Dia-Deceleration [%]	AUC of positive force during early diastole	Like (6), summation limited to that phase of diastole

There is significant variability of strain measurements between different software, which may affect the generalizability of clinical outcomes of data analyzed by feature-tracking (42). Since HDF are derived from strain measurements, inter-vendor differences in CMR feature-tracking software packages may affect the generalizability of HDF data (43). Prior studies have demonstrated that myocardial deformation metrics derived from feature-tracking CMR are highly dependent on software implementation. Bourfiss et al. showed that right ventricular peak longitudinal strain assessed using four commercially available feature-tracking CMR software packages exhibits heterogeneous feasibility and poor agreement in absolute values, with wide limits of agreement between methods (44). Similarly, Schuster et al. reported that myocardial strain and torsion could be reliably detected during dobutamine stress using two different commercial CMR feature-tracking tools, yet both inter- and intravendor variability remained substantial, even after averaging multiple analyses (45). All together, these findings highlight that while feature-tracking-derived metrics are reproducible at a group level, methodological differences across software packages may lead to disparate absolute values, a limitation that is likely to affect HDF calculations derived from strain data. Future standardization of software must be implemented in order to consolidate the adoption of cine CMR-derived HDF in clinical and research settings.

## 9 Potential future developments in the field

Automated analysis of HDF curves using clustering techniques has been recently proposed (46). This automatic approach is promising to analyze the entire time course of both longitudinal and transversal components of the HDF, which avoids the description of one or a few parameters of HDF curves. When further extended and validated, this approach may facilitate the analysis of HDF curves, categorize patients according to the disease severity, and help providers in planning personalized treatment (47).

## 10 Conclusions

HDF curves display the amplitude and direction of the IVPG during a cardiac cycle. Data can be derived from CMR images; therefore, the scanning protocol and the quality of image planning are crucial for the accuracy of HDF. The application of a mathematical model has made HDF analysis feasible with cine CMR, irrespective of the availability of 4D flow, expanding the clinical applicability of this method.

Once properly assessed, the analysis of HDF curves can be useful in various clinical scenarios for the detection of subclinical LV dysfunction, proper follow-up, and prediction of future cardiac events. In athletes, HDF analysis may provide additional insight into physiological vs. pathological cardiac remodeling and contribute to more refined cardiovascular screening and monitoring strategies.
